# Psychometric properties of the International Trauma Questionnaire (ITQ) examined in a Norwegian trauma-exposed clinical sample

**DOI:** 10.1080/20008198.2020.1796187

**Published:** 2020-08-14

**Authors:** Peter Sele, Asle Hoffart, Harald Bækkelund, Tuva Øktedalen

**Affiliations:** a Research Institute, Modum Bad, Vikersund, Norway; b Psychological Institute, University of Oslo, Oslo, Norway; c Norwegian Center for Violence and Traumatic Stress Studies, Oslo, Norway; d Center of Expertise for Parental Support and Prevention, Children, Youth and Family Directorate, Oslo, Norway

**Keywords:** generalizability, complex post-traumatic stress disorder (CPTSD), International Trauma Questionnaire (ITQ), post-traumatic stress disorder (PTSD), confirmatory factor analysis, ICD-11, reliability, validity, generalización, Trastorno de Estrés Postraumático Complejo (TEPT-C), Cuestionario Internacional de Trauma (ITQ), Trastorno de Estrés Postraumático (TEPT), Análisis Factorial Confirmatorio, CIE-11, Confiabilidad, Validez, 概化性, 复杂性创伤后应激障碍 (CPTSD), 国际创伤问卷 (ITQ), 创伤后应激障碍 (PTSD), 验证性因子分析, ICD-11, 可靠性, 有效性, • The validity of the Norwegian translation of the International Trauma Questionnaire (ITQ) is supported.• Test users are encouraged to take the particular purpose of their test use into account when choosing an appropriate format of the ITQ.

## Abstract

**Background:**

The International Trauma Questionnaire (ITQ) is a self-report measure for post-traumatic stress disorder (PTSD) and complex post-traumatic stress disorder (CPTSD), corresponding to the diagnostic criteria in the International Classification of Diseases, 11th Revision (ICD-11). A 12-item version of the ITQ based on samples from English-speaking countries has been presented, and the wider generalizability to other languages needs to be examined.

**Objective:**

The current study examines the psychometric properties of scores from a longer, preliminary 22-item version of the ITQ and the current reduced 12-item version by means of generalizability theory (G-theory) and confirmatory factor analysis (CFA).

**Method:**

The 22-item version of the ITQ was translated into Norwegian and administered to patients in two trauma treatment trials (total *N* = 202). A generalizability study was used to investigate the psychometric properties of scores reflecting CPTSD. G-theory was also used to investigate alternative measurement designs to optimize the sufficient number of items that provide acceptable generalizability and dependability of scores. Model fit to the theoretical factor structure was then examined by CFA, both for the 22-item version and for the 12-item version of the ITQ.

**Results:**

The two subscales negative self-concept and relational disturbances had acceptable generalizability coefficients. We found substantial measurement error related to affective dysregulation, mainly attributable to affective hyperactivation. A latent factor structure model with two separate affective dysregulation factors: hyperactivation and deactivation, represented the data well in the 22-item version. The proposed confirmatory structure model for the 12-item short form did not converge in the CFA.

**Conclusion:**

This study supports the applicability of the ITQ in a non-English-speaking country and provides support for the validity of the Norwegian translation. Further research is needed to improve the psychometric properties of the affective dysregulation subscale.

## Abstract (es)

**Antecedentes**: El Cuestionario Internacional de Trauma (ITQ en su sigla en inglés) es una medida de autoreporte para el trastorno de estrés postraumático (TEPT) y el trastorno de estrés postraumático complejo (TEPT-C), que corresponde a los criterios de diagnóstico de la CIE-11. Recientemente, se presentó una versión de ITQ de 12 ítems basada en muestras de países de habla inglesa, y es necesario examinar la posibilidad de generalización a otros idiomas.

**Objetivo**: El presente estudio examina las propiedades psicométricas de los puntajes de una versión preliminar más larga de 22 ítems del ITQ y la versión reducida actual de 12 ítems por medio de la Teoría de la Generalización (TG) y el Análisis Factorial Confirmatorio (AFC).

**Método**: La versión de 22 ítems de ITQ fue traducida al noruego y fue administrada a los pacientes en dos ensayos de tratamiento de trauma (total *N* = 202). Se usó un estudio de Generalización para investigar las propiedades psicométricas de las puntuaciones que reflejan el TEPT-C. La TG también se usó para investigar los diseños de medición alternativos para optimizar el número de ítems suficientes para proporcionar una generalización y confiabilidad aceptables de los puntajes. El ajuste del modelo a la estructura factorial teórica se examinó mediante un análisis factorial confirmatorio, tanto para la versión de 22 ítems como para la versión de 12 ítems del ITQ.

**Resultados**: Las dos subescalas de autoconcepto negativo y la de perturbación en las relaciones tenían coeficientes de generalización aceptables. Encontramos un error de medición sustancial relacionado con la desregulación afectiva, principalmente atribuible a la hiperactivación afectiva. Un modelo de estructura factorial latente con dos factores de desregulación afectiva separados, hiperactivación y desactivación, representó bien los datos en la versión de 22 ítems. El modelo de estructura confirmatoria propuesto para el formato corto de 12 ítems no convergió en el análisis AFC.

**Conclusión**: El presente estudio respalda la aplicabilidad de ITQ en un país de habla no inglesa, y brinda soporte para la validez de la traducción al noruego. Se necesita más investigación para mejorar las propiedades psicométricas de la subescala de desregulación afectiva.

## Abstract (zh)

**背景**: 国际创伤问卷 (ITQ) 是对创伤后应激障碍 (PTSD) 和复杂性创伤后应激障碍 (CPTSD) 的自评式测量, 与ICD-11诊断标准相对应。最近提出了一个基于英语国家样本的12条目版ITQ, 其广泛应用于其他语言的概化性有待探究。

**目的**: 本研究通过概化理论 (GT) 和验证性因子分析 (CFA), 考查较长的初始22条目ITQ以及当前12条目缩减版得分的心理测量学特性。

**方法**: 在两次创伤治疗试验中 (总计*N* = 202), 将22条目版ITQ翻译成挪威语并施用于患者。概化研究用于探究反映CPTSD得分的心理测量学特性。 G理论也用于探究替代测量设计, 以优化提供可接受概化性和可靠性得分的条目数量。然后用验证性因子分析考查ITQ的22条目版和12条目版模型与理论因子结构的拟合。

**结果**: 两个分量表的负性自我概念和关系障碍具有可接受的概化系数。我们发现情感失调相关的大量测量误差主要归因于情感过度激活。具有两个独立情感失调因子 (过度激活和失活) 的潜在因子结构模型很好地代表了22条目版的数据。为12条目简短形式提出的验证性结构模型在CFA分析中不收敛。

**结论**: 当前研究支持ITQ在非英语国家的适用性, 并为挪威语翻译的有效性提供支持。需要进一步研究以提高情绪失调分量表的心理测量学特性。

## 1. Introduction

The International Classification of Diseases, 11th Revision (ICD-11) working group on disorders specifically associated with stress distinguishes complex post-traumatic stress disorder (CPTSD) from post-traumatic stress disorder (PTSD) (World Health Organization, 2019). PTSD and CPTSD share the gate criteria of exposure to one or more potentially traumatic events. PTSD is defined by the presence of re-experiencing symptoms (Re), such as intrusive nightmares and flashbacks of the event, avoidance (Av) of internal and external stimuli associated with the trauma, and a sense of current threat (Th). Alongside these symptoms, persons with CPTSD also suffer from problems with affect dysregulation (AD), negative self-concept (NSC) and disturbances in relationships (DR) related to their trauma. These last three symptom clusters are jointly referred to as disturbances in self-organisation (DSO) (Maercker et al., 2013).

The term ‘complex PTSD’ was first coined by Judith Herman (1992) to fit the varying symptoms and difficulties encountered by survivors of repeated and prolonged interpersonal trauma. Studies have found that both childhood abuse and war captivity in adulthood are linked to an increased risk for CPTSD (Hyland et al., 2017; Zerach, Shevlin, Cloitre, & Solomon, 2019).

Further research on CPTSD, its prevalence and treatment implications, rests on the availability of valid and reliable diagnostic instruments to assess and differentiate PTSD and CPTSD in various languages and cultural contexts. The International Trauma Questionnaire (ITQ) has been developed to be an accessible and usable self-report measure (Cloitre et al., 2018). The ITQ is available in a number of translations (e.g. Bondjers & Arnberg, 2015; Ho et al., 2019; Kazlauskas, Gegieckaite, Hyland, Zelviene, & Cloitre, 2018; Vallières et al., 2018). The instrument is constructed to reflect two overarching constructs, PTSD and DSO. Within PTSD and DSO, items are nested in six subordinate symptom clusters. Studies of the ITQ have found the internal reliability of the six ITQ symptom clusters to be acceptable in both clinical (Cloitre et al., 2018; Karatzias et al., 2016) and non-clinical samples (Ben-Ezra et al., 2018; Cloitre et al., 2018; Ho et al., 2019). In factor analytic studies, two structural representations of the ITQ have repeatedly gained support. A correlated six-factor (Re, Av, Th, AD, NSC and DR), first-order model seems to fit the data best in trauma-exposed community (Cloitre et al., 2018) and student samples (Ho et al., 2019). In clinical samples, a correlated, second-order model closely corresponding to the ICD-11 diagnostic taxonomy has been found to be superior. In this model, PTSD explains the covariation between Re, Av and Th; and DSO the covariation between AD, NSC and DR (Cloitre et al., 2018; Karatzias et al., 2016). It is worth noting that the differences between the two models are modest in most studies. Ben-Ezra et al. (2018) found support for a third structural model in an Israeli trauma-exposed community sample. In this model, affect dysregulation is split into two separate hyperactivation and deactivation factors, but is otherwise equal to the second-order model above. To our knowledge, this model has not been examined in clinical samples.

Initial studies of the ITQ used versions with six or more items to capture PTSD and 16 items to capture DSO (Karatzias et al., 2016; Kazlauskas et al., 2018). To ensure ease of administration and scoring while preserving the core symptoms of CPTSD, the developers of the ITQ aimed to reduce the number of DSO items. Shevlin et al. (2018) concluded that all the DSO items measured their intended symptom clusters well, and a final 12-item version of the ITQ with six DSO items has been proposed (Cloitre et al., 2018). The development of test versions with fewer items is typically motivated by a reduction of the burden on the respondent, or a test administrator’s wish to cover more concepts within a restricted period. Reduction of a test form to a more limited set of items requires several considerations, among them the implications for influence of measurement error on the test results. Is reliable assessment of person-related differences maintained in a short form? The ITQ is a complex measure, and the number of items needed to assess each symptom cluster reliably may vary. In addition, the items in the ITQ reflect various latent constructs defined by theory. Are the intended construct domains adequately covered with fewer items? Both questions relate to the dependability of the ITQ scores and, to our knowledge, they have not been addressed in prior studies of the ITQ short form.

The implications of item reduction for the practical utility of the ITQ also warrant consideration. Kane (2001) discusses the role of consequences in test validation, and proposes a distinction between the set of inferences leading from test scores to statements about persons, and decisions based on these statements. Although the ITQ primarily has been presented as a diagnostic screening tool (Cloitre et al., 2018), it is conceivable that future practical use could involve other clinical decisions and purposes. These include categorization of persons as in need of treatment intervention or not with reference to a clinical cut-off point, rank ordering of patients by symptom severity (as a basis for prioritizing certain treatment interventions for certain patients) or feedback to individual patients on how their symptoms change during treatment. The validity of such forms of use will rest both on the dependability of the ITQ scores and on the test user’s familiarity with the strengths and limitations of the ITQ for different decision-making purposes.

In the present study, we aimed to examine the psychometric properties of scores from both the longer, preliminary 22-item version of the ITQ and the current reduced 12-item version, in a Norwegian clinical sample. Specifically, we examined reliability and validity in terms of generalizability and model fit to the theoretical structural model. Analyses based on generalizability theory (G-theory) and confirmatory factor analysis (CFA) were used.

## 2. Methods

### 2.1. Participants and procedures

Study participants were patients from two ongoing Norwegian trauma treatment studies. The first is a randomized controlled trial of outpatient stabilizing group treatment (*N* = 152) and the second is an ongoing randomized controlled trial comparing prolonged exposure, skills training in affective and interpersonal regulation (STAIR) and STAIR + narrative therapy (NT) in an inpatient setting (*N* = 50). In both studies, a local physician, psychologist or psychiatrist had referred the participants to specialized trauma treatment prior to recruitment. Data were collected at pretreatment assessment in the first study and at treatment start in the second study. Both studies have been approved by the Regional Committees for Medical and Health Research Ethics, Health South-East.

The total sample consisted of 202 patients, with a mean age of 41.5 years (SD = 9.5, range = 24–69 years). Of these, 53.1% were married or living with a partner in a committed relationship, 28.2% were employed full or part time, 7.9% were students and 70.3% received full or partial welfare benefits (e.g. sick leave, disability pension). Exposure to interpersonal trauma in childhood was assessed by the Childhood Trauma Questionnaire in Study 1 (Bernstein & Fink, 1998; Dovran et al., 2013) and Stressful Life Events Screening Questionnaire in Study 2 (Goodman et al., 1998; Thoresen & Øverlien, 2009), and is reported in Table 1. Almost all patients reported more than one type of trauma (92%). By the ITQ’s latest diagnostic algorithm (Cloitre et al., 2018), a minority of the sample (13.4%) met the requirements for PTSD while over half of them (60.4%) met criteria for the CPTSD diagnosis. The remaining patients (26%) had substantial symptoms without reaching full diagnostic criteria for either disorder. Out of the six PTSD and DSO symptom clusters, the mean number of symptom clusters endorsed by this group was 3.9 (SD = 1.2). The estimated diagnostic rates are based on symptom items only.

**Table 1.** Sample characteristics: gender, age, diagnosis and exposure to interpersonal childhood trauma.

Variable	Study 1 (*N *= 152)	Study 2 (*N* = 50)	Total (*N* = 202)
Female	86.2% (131)	80% (40)	84.7% (171)
Age (years), mean (SD)	41.3 (9.9)	42.2 (8.5)	41.5 (9.5)
PTSD	15.8% (24)	6.2% (3)	13.4% (27)
CPTSD*	59.9% (91)	62.0% (31)	60.4% (122)
Emotional abuse	92% (140)	68% (34)	86.1% (174)
Sexual abuse	76% (116)	80% (40)	77.2% (156)
Physical abuse	60% (91)	72% (36)	62.9% (127)

*According to ICD-11, a person can only receive a post-traumatic stress disorder (PTSD) or a complex post-traumatic stress disorder (CPTSD) diagnosis, not both.

### 2.2. Measures

#### 2.2.1. PTSD and DSO symptoms

Both studies used a Norwegian translation of the preliminary 22-item version of the ITQ. Three experienced clinicians separately translated the measure from English to Norwegian, discussed discrepancies and reached consensus on a final translation. A separate, bilingual psychologist translated the Norwegian version back to English. One of the original authors of the ITQ approved the back-translation. The 12-item version of the Norwegian ITQ is publicly available (Bækkelund, Sele, & Berg, 2019).

The first section of the 22-item ITQ is devoted to three PTSD symptom clusters: re-experiencing of the trauma, avoidance of internal or external trauma reminders, and sense of current threat. These are measured by two items each (Re1 and Re2, Av1 and Av2, and Th1 and Th2). The second section consists of 16 DSO items. DSO is subdivided into three main symptom clusters: affective dysregulation (both hyperactivation and deactivation) (AD1–AD9), negative self-concept (NSC1–NSC4) and disturbances in relationships (DR1–DR3). All items are answered on a five-point Likert scale, ranging from ‘Not at all’ to ‘Extremely’. In the PTSD section, respondents are instructed to report how much they have been bothered by the symptom in the past month. For DSO symptoms, they are asked to report how they typically feel, think about themselves and relate to others.

The current ITQ version has six PTSD items, six DSO items and three functional impairment items related to each symptom category (Cloitre et al., 2018). The six items chosen to represent the three DSO clusters are AD2 and AD6, NSC1 and NSC2, and DR1 and DR2 from the version we used. Functional impairment items were not part of the ITQ version used in this study, and the reported diagnostic rates are based on the 12 symptom criteria alone.

In the present study, the complete 22-item ITQ was used in the first study (both PTSD and DSO items, *N* = 152). In the second study (*N* = 50), participants completed the 16 DSO items of ITQ. The remaining six PTSD items were collected from corresponding items in the PTSD Checklist for DSM-5 (PCL-5) (Weathers et al., 2013). The PCL-5 items are reported on the same five-point Likert scale (with slight semantic differences in anchors), and used to construct a complete ITQ score.^1^ See Table A1 in the Appendix for descriptive statistics and item endorsement rates (i.e. items scored ≥ 2).

### 2.3. Statistical analysis

G-theory is a formal statistical approach suited for investigating the psychometric properties of scores in multi-facet measurement designs, like the ITQ. It is applicable when the aim is to optimize a measure by reducing measurement error and the number of items without narrowing the construct domain (Brennan, 2011). Studies based on G-theory provide estimates of how the dependability of scores changes as the number of items changes in different test formats. This can aid a test designer’s decision about the appropriate number of items in a new test format. Thus, G-theory supplements other analytic strategies in short-form development (e.g. item response theory or CFA) more suited to select the specific items to include in a new test format. Two types of studies are conducted within G-theory: generalizability studies (G-studies) and decision studies (D-studies).

A G-study provides information on the different variance components of a test. Both variance related to the intended object of measurement (variance in test scores that is attributable to differences between persons) and various sources of measurement error are estimated. Sources of measurement error can be differences related to items, raters or test occasions.

A D-study uses information from a G-study to design the best possible application of a measurement for a particular purpose (Webb, Shavelson, & Haertel, 2006). A distinct characteristic of G-theory is the distinction made between reliability involving absolute decisions, which is relevant if clinical decisions are based on an individual’s score, and relative decisions involving stability in relative standing or rankings of persons (Brennan, 2003; Feldt & Brennan, 1989). This distinction is important and needed in clinical practice because most clinical decisions concern the standing of a given patient with regard to criteria used for determining clinical intervention (absolute decisions). In G-theory, the term ‘universe score’ refers to the long-run average of observed scores a person would obtain in the broad universe of admissible observations, analogous to ‘true score’ in classical test theory. Two types of relevant coefficients can be estimated to represent different definitions of measurement error: the ‘generalizability coefficient’ (G-coefficient) is the ratio of universe score variance to itself plus relative error variance, and the ‘index of dependability coefficient’ is a more conservative estimate of reliability, defined by the ratio of the universe score to itself plus absolute error variance. G-coefficients > .80 are regarded as acceptable. A total of six facets (including the object of measurement) may be estimated simultaneously in balanced designs. Multivariate G-study and D-study analyses were conducted in mGENOVA (Brennan, 2001).

First, we examined the 22-item ITQ in a multi-facet G-study (*p* × *i* design). We treated the DSO symptom clusters (affective dysregulation, negative self-concept and relational disturbances) as three separate fixed facets and the three PTSD symptom clusters (re-experiencing, avoidance and sense of current threat) as one fixed facet.^2^ Items within each fixed facet were regarded as randomly selected indicators and treated as a random facet. Three sources of measurement variance (persons, items, and person by items interactions) were estimated separately for the four fixed facets. The person component is the intended object of measurement, and reflects variance related to individual differences. The item component reflects measurement error related to systematic inconsistencies between items in a facet, across persons. G-theory is a random sampling theory and, as such, items are assumed to be randomly sampled from an infinite universe of items that are equivalent representations of a latent construct. The item component represents the degree of violation of this assumption. Item by person interaction is a second source of measurement error. It provides estimates of variation in the rank ordering of individuals based on different items. Acceptable scores are indicated by a combination of a high person component and low error components (item component, and item by person interaction component).

Secondly, we conducted a D-study of the 22-item ITQ to obtain a composite G-coefficient for the test as a whole, and separate G-coefficients for PTSD, affective dysregulation, negative self-concept and relational disturbances. We repeated these analyses to obtain the same information using the items included in the 12-item form proposed by Cloitre et al. (2018).

**Figure A1.**
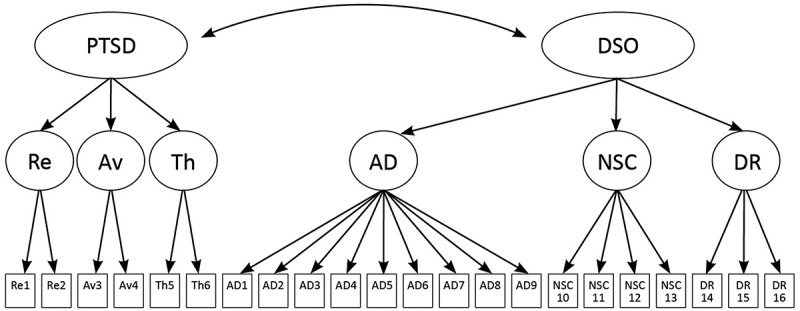
Model 1, two-factor second order model with affective dysregulation as one factor.

**Figure A2.**
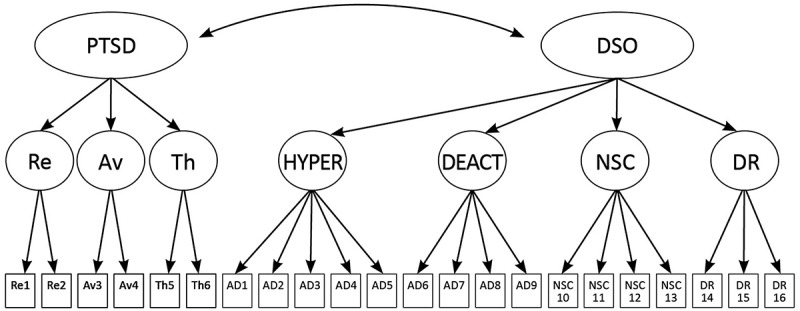
Model 2, two factor second order model with affective dysregulation as two factors.

**Figure A3.**
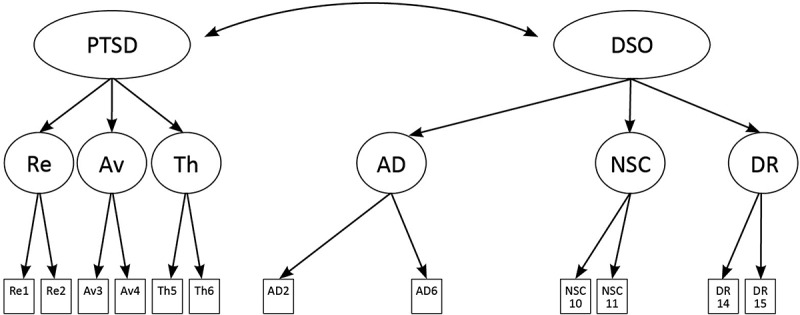
Model 1, based on the 12 items ITQ.

Thirdly, we analysed factor structure in the ITQ by comparing two previously proposed models in CFA. Model 1 (Figure A1) closely corresponds to the ICD-11 proposal, with two correlated second-order factors (PTSD and DSO), each with three underlying first-order factors (for PTSD: Re, Av and Th; and for DSO: AD, NSC and DR) (Cloitre et al., 2018). In model 2 (Figure A2), affect dysregulation is construed as two separate factors, affective hyperactivation and deactivation, both loading on DSO (Ben-Ezra et al., 2018). The analysis of model 1 was repeated for the 12-item short form developed by Cloitre et al. (2018) to assess factorial stability (Figure A3). See Appendix for a graphical presentation of the models.

We used the means and variance-adjusted weighted least squares (WLSMV) estimator for the CFA analyses. WLSMV provides accurate parameter estimates, standard errors and test statistics for ordinal indicators. The amount of missing data was low, with 61 missing data points (1.3%). Standard criteria were used to assess model fit. Comparative fit index (CFI) and Tucker–Lewis index (TLI) values ≥ 0.90 indicated acceptable fit, and values ≥ 0.95 indicated excellent fit; root mean square error of approximation (RMSEA) values ≤ 0.8 indicated acceptable fit and values ≤ 0.5 indicated excellent fit (Hu & Bentler, 1999). WLSMV does not produce information-based indices needed for comparisons of model fit. Therefore, all models were also fitted using robust maximum likelihood (MLR) to obtain the Bayesian information criterion (BIC). A model is considered to have strong evidence of statistical superiority when BIC values are 6–10 points lower than a competing model (Raferty, 1995). Mplus 8 was used in all CFA analyses (Muthen & Muthen, 2015).

## 3. Results

### 3.1. G-study

In an initial analysis, we found high estimates of measurement error in the affective dysregulation scale. The estimates for person-related variance and item-related variance were .406 and .467, respectively. This means that the observed scores are more strongly related to measurement error than to the intended object of measurement, person differences. Based on this result, we chose to split the affective dysregulation facet into two forms of emotional problems embedded in the scale, which are termed hyperactivation and deactivation. This allowed for separate estimates of variance related to persons and measurement error in the two scales. For deactivation, we found a high person component and a low item component. Person by item interaction was high, reflecting differences in rank ordering of persons by different items. For the hyperactivation scale, the person component was low and item-related variance was high, indicating a high degree of measurement error for items of this particular subscale.

Negative self-concept showed satisfactory scores with a high person component relative to a low item component. Relational disturbances showed the same desired pattern of high person component and low item component. Error variance related to person by item interaction was also lower than the person component in both facets. In sum, this reflects that variances of scores in negative self-concept and relational disturbances are systematically related to individual differences, with little influence of measurement error. For PTSD, the person component was also higher than the item component, but lower relative to person by item interaction, reflecting differences in rank ordering of individuals from different items in this subscale.

The G-study results for the facets negative self-concept and relational disturbances indicate that item reduction is viable without compromising the dependability of the scores. Fewer items may probably measure affective deactivation adequately, too. Item reduction seems less feasible for affective hyperactivation owing to extensive item- and item by person-related variance. Results from the G-study are reported in Table 2.

### 3.2. D-study

The D-study estimates for the 22-item version and a 12-item short form version of the ITQ are displayed in Table 3.

**Table 2.** Estimated G-study variance and covariance components for 22-item International Trauma Questionnaire based on *p* × *i* design (*N* = 165).

Source	PTSD	Hyperactivation	Deactivation	Negative self-concept	Disturbance in relationships
Persons (*p*)	.43300	.29041	.78339	1.04739	.90989
Items (*i*)	.06423	.88118	.05910	.00803	.03130
Persons by items (*pi*)	.90527	.88851	.85201	.37430	.53436
Relative contribution to composite universe score	22.19%	16.11%	22.03%	22.31%	17.36%

PTSD, post-traumatic stress disorder.

**Table 3.** D-study estimates for International Trauma Questionnaire versions with 22 and 12 items.

	Number of items = 22	Number of items = 12
*σ*^2^	.42226	.41682
*σ*^2^ (*δ*)	.03385	.06389
*σ*^2^ (Δ)	.04450	.07361
*Eρ*^2^	.92579	.86709
Φ	.90467	.84992

*σ*^2^, universe score variance; *σ*^2^ (*δ*), relative measurement error; *σ*^2^ (Δ), total measurement error; *Eρ*^2^, generalizability coefficient; Φ, index of dependability.

For the 22-item version of the ITQ, the composite G-coefficient score was excellent (.926). The G-coefficients of the facets displayed more variation. PTSD was marginally lower than desired at .74, hyperactivation was substantially lower than desired at .62, deactivation was acceptable at .79, negative self-concept was excellent at .92 and relational disturbances was acceptable at .84. The 12-item short form version had a lower, but still acceptable, composite G-coefficient (.86709). The G-coefficient estimates for the subscales negative self-concept, .85, and relational disturbances, .77, were both acceptable. However, the affective dysregulation scale had a very low G-coefficient value of .45.

### 3.3. CFA

Two confirmatory factor structure models were compared for the 22-item ITQ (see Figures A1 and A2 in the Appendix). Model 1 has a single affect dysregulation factor loading on DSO. Model 2 distinguishes between hyperactivation and deactivation as two separate affect dysregulation factors. Both models broadly correspond to the theoretical structure of CPTSD in ICD-11. Both models 1 and 2 have acceptable goodness-of-fit indices, with chi-squared to degrees of freedom ratios < 3:1, RMSEA levels < .08 and CFI/TLI > 0.95 (Table 4). Comparing BIC and sample-size adjusted BIC values indicates strong evidence of statistical superiority of model 2 to model 1 (Raferty, 1995). This suggests that model 2 should be retained, despite being more complex than model 1. Model fit statistics are displayed in Table 4.

**Table 4.** Comparison of two models of the International Trauma Questionnaire (*N* = 202).

Model	Items	Chi-square (df)	RMSEA (90% CI)	CFI	TLI	BIC	ssaBIC
1	22	437.473* (202)	.076 (.066–.086)	.963	.957	12457.606	12226.328
2	22	402.833* (201)	.071 (.060–.080)	.968	.963	12426.130	12191.683

RMSEA, root mean square error of approximation; CI, confidence interval; CFI, comparative fit index; TLI, Tucker–Lewis index; BIC, Bayesian information criterion; ssaBIC, sample-size adjusted Bayesian information criterion.

*All *p*-values are ≤ .001.

Standardized factor loadings for model 2 are displayed in Table A2 (Appendix). All first-order loadings on re-experiencing (Re), avoidance (Av), threat (Th), negative self-concept (NSC) and disturbed relationships (DR) were positive, high (> .70) and significant (*p *< .001). The factor loadings for the two parts of affective dysregulation (AD) differed. Affective deactivation had positive and high loadings for all items (> .70). For affective hyperactivation, one item had a satisfactory factor loading above .70, three items loaded between .50 and .70, and one item (reckless behaviour) had a low loading of .29. The second-order loadings of hyperactivation, deactivation, negative self-concept and relational disturbances on DSO were all high (> .70) and statistically significant. For the PTSD factor, the second-order factor loading for threat was high (.88). Re-experiencing (.68) and avoidance (.61) had lower, but acceptable, loadings on PTSD. The two second-order factors, PTSD and DSO, were highly correlated (*r *= .81, *p* < 0.001).

The CFA of model 1 based on the 12 items proposed for the ITQ short form (Cloitre et al., 2018) did not converge. We found negative residuals in the AD factor. Two alternative models were tested. Neither a model allowing the hyperactivation item of AD to load on threat in addition to AD, nor a model where AD loaded on both PTSD and CPTSD resulted in converging models. Cloitre et al. (2018) used dichotomized variables in their study. Recoding our data set to dichotomized variables did not provide converging solutions. Problems with non-converging solutions may result from overparameterization, as each of the six first-order factors rests on only two indicators.

### 3.4. Supplementary analysis

We set out to examine the underlying structure of the 12-item short form. The results from the CFA indicated the need for supplementary data analysis to suggest an alternative short form. New D-studies were performed and compared to find a model with acceptable dependability estimates. An 18-item design with six PTSD items, five hyperactivation items, three deactivation items, and two items each for negative self-concept and relational disturbances was suggested to provide the best balance between brevity and dependability of the scores. The composite G-coefficient for this measurement design was acceptable (.897). The G-coefficients for the separate hyperactivation and deactivation scales were markedly improved compared to the poor G-coefficient (.45) found for the joint affect dysregulation scale in the 12-item design. However, the hyperactivation subscale was unchanged from the 22-item design, and still had a low G-coefficient of .62. The G-coefficient of .73 for the deactivation scale was also somewhat lower than desired.

D-studies give information on the appropriate number of items needed for reliable estimation of each facet, but not on the selection of specific items. To reduce the number of items to an 18-item short form, we used CFA and inspected the standardized factor loadings of the items in the 22-item version (see Table A2 in the Appendix). Items with high loadings (> .70) on their corresponding factor and low cross-loadings to other factors (as indicated by inspection of modification indices) were considered as candidates for a short form. The modest G-coefficient in the D-study indicated that all five hyperactivation items should be retained (AD1–AD5). To represent deactivation, AD6, AD8 and AD9 were selected. AD7 also loaded adequately (< .70) on deactivation, but had high cross-loadings to both NSC and DR. For negative self-concept, both NSC10 and NSC11 had high loadings on the factor and low cross-loadings to other factors. For relational disturbances, both DR14 and DR15 had high factor loadings and low cross-loadings to other factors. While the AD items are changed, the PTSD, NSC and DR items are the same as in the 12-item version.

**Figure A4.**
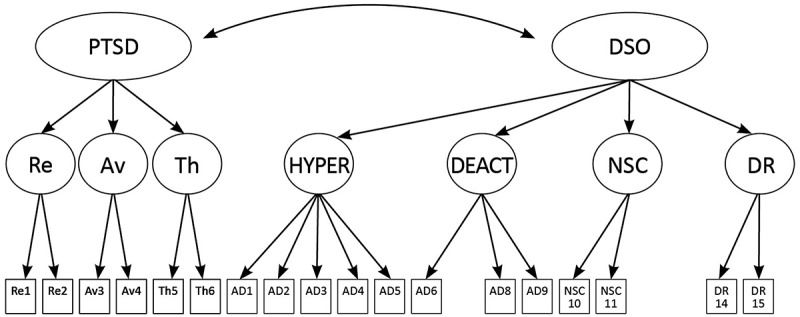
Model 2, based on the 18 items ITQ.

We then examined the factorial stability of model 2 in this 18-item version, and found acceptable model fit indices: χ^2^ (127) 238.935, *p* < .05, RMSEA = .066 (90% confidence interval = .053–.079), CFI = .974 and TLI = .969. See Figure A4 in the Appendix for a graphical presentation.

## 4. Discussion

The ITQ provides clinicians and researchers with the first instrument to assess PTSD and CPTSD in line with ICD-11 diagnostic criteria. This study is based on a Norwegian translation of the instrument and adds to an expanding set of data about the psychometric properties of scores from the ITQ.

By means of generalizability theory, this study contributes to a further understanding of the sources of measurement variance and measurement error in the ITQ. Test scores inevitably reflect both an intended object of measure (in this case, individual differences in CPTSD symptoms) and other unintended variance components with the potential to reduce dependability of the scores. Estimates of these variance components provide important information when the aim is to develop reliable short-form versions.

For PTSD symptoms, we found that both when the three symptom clusters (Re, Av and Th) were estimated as one facet and when they were estimated separately (see Table A3 in the Appendix), person-related variance was high and item-related error was modest. The larger person by item interaction component indicates that the rank ordering of persons may differ with different PTSD items. The overall G-coefficient was acceptable, although in the lower range.

We found high estimates of person-related variance and little measurement error for two of the three DSO facets, namely negative self-concept and relational disturbances. The G-coefficients for these two facets were still acceptable when the number of items was reduced to two, supporting the proposal in the 12-item ITQ (Cloitre et al., 2018).

For the third DSO cluster, affective dysregulation, we found problematic measurement error estimates, with scores reflecting item-related variance and person by item-related variance to a larger extent than person-related variance. The affect dysregulation facets with two items, parallel to the 12-item ITQ (Cloitre et al., 2018), had a low G-coefficient. From a psychometric perspective, this calls for refinement of the facet, possibly by adding items to reduce the influence of measurement error to more adequately target the construct domain.

This study also suggests that affective dysregulation in the ITQ may be more properly conceived as two different facets than as one facet. Separate analysis of the variance components for hyperactivation and deactivation gave valuable information on the sources of the problematic error estimates. Deactivation had high person-related variance and acceptable error estimates, while hyperactivation had the opposite pattern (low person-related variance and high error estimates). The G-coefficient for hyperactivation (with all five items retained) was clearly below the acceptable level, with a value of .62. Based on these findings, further item reduction on the hyperactivation facet would not be recommended. Regarding the deactivation facet, the G-coefficient estimates were less conclusive. It is debatable whether three items yield sufficient dependability of scores, or if all four items should be retained.

The confirmatory factor analyses of two structural models of the ITQ, model 2 with a split affective dysregulation factor (where hyperactivation and deactivation are seen as separate factors) and model 1 with a merged affective dysregulation factor, contribute to the same picture. A BIC difference above 30 points favours the split model over the merged model. This split model is not among the most frequently studied models, but our finding replicates the findings from a trauma-exposed community sample (Ben-Ezra et al., 2018) in a clinical sample of childhood abuse patients. This strengthens the argument for a differentiated view on affective dysregulation.

Affective dysregulation is a central problem area in CPTSD and theoretically complex. Both undermodulated and overmodulated affect are repeatedly found to be common consequences of trauma (Lanius, Brand, Vermetten, Frewen, & Spiegel, 2012) and the ITQ is intended to cover both forms (Cloitre et al., 2018). Theories of emotion dysregulation, e.g. the ‘window of tolerance’ model, propose that these forms of dysregulation are closely associated in persons exposed to interpersonal childhood trauma. Siegel (2012) states that repeated exposure to out-of-control emotions in childhood, combined with the lack of effective caregiver regulation, develops into impairments in the ability to self-soothe effectively in adulthood. Consequentially, later emotionally challenging experiences may overwhelm the person’s regulatory capacity, resulting in frequent states of hyperactivation or deactivation, or oscillation between the two. Thus, different forms of affect dysregulation problems are expected to vary across individuals and over time within individuals. Both our results and other studies based on the ITQ suggest that we do not know the precise relation between hyperactivation and deactivation problems. A study of CPTSD symptom networks found high interrelatedness of symptoms (nodes) in negative self-concept and relational disturbances, but weaker associations within the symptoms of affective dysregulation (Knefel et al., 2019). Previous factor analytic studies also give a mixed picture. Karatzias et al. (2016) found weak factor loadings (< .60) for seven out of nine items on the affect dysregulation scale. Hyland et al. (2017) report higher factor loadings, > .70 for six out of nine items, whereas Rocha et al. (2019) propose that affect dysregulation should be split into three different factors. The samples in the above studies vary in the extent and type of traumatic experiences. In our study, the majority of the participants had been exposed to severe, repeated interpersonal childhood trauma. In that aspect, the affect dysregulation problems they report could be expected to approach Siegel’s (2012) description. Our findings suggest that dominance of either hyperactivation or deactivation symptoms may be a more common clinical presentation than a pattern of frequent shifts between the two. A more thorough understanding of the relation between hyperactivation and deactivation may have important implications for treatment and should be a focus for further studies.

A further important finding from this study is the inability to replicate the structure model for the 12-item short form. While Cloitre et al. (2018) uses dichotomized variables (symptoms scored ≥ 2 are regarded as present), we used the full five-point scale. However, this difference did not account for the non-converging CFA models in our study. The theoretical structural model for the 12-item ITQ is complex. With only two indicators reflecting each of the six first-order factors and two correlated second-order factors, there is a risk of overparameterization of the model.

The ITQ is a self-report measure that provides operational definitions of the ICD-11 criteria for PTSD and CPTSD. We found acceptable reliability estimates for two of the three DSO facets in the 12-item short form. Our finding suggests that the affective dysregulation scale needs further refinement. Regarding hyperactivation and deactivation separately is a viable alternative to a merged facet. We found that three items provided dependable estimates of deactivation, while hyperactivation needed five (or preferably more) items.

At this time, the ITQ is the only available self-report measure of CPTSD that corresponds directly with the ICD-11 criteria, and therefore it is valuable in a variety of contexts. The 18-item short form adds to the list of ITQ versions, potentially expanding the applicability of the ITQ to areas beyond diagnostic screening, such as patient feedback on symptom change during therapy, or treatment decisions based on an individual’s standing compared to clinical cut-off points. These applications of the ITQ involve interpretation of the test results of individual patients. The validity of such interpretations rests on the use of a measurement design with acceptable dependability estimates for the particular test purpose. This consideration should be taken into account when researchers and clinicians decide on the appropriate format of the ITQ for their use. It remains debatable whether an 18-item version is a substantial reduction of respondent burden compared to using the full 22-item form. Both the 18-item and the 22-item versions provide test users with alternative formats, thus expanding the practical utility of the ITQ.

This study has some limitations. A majority of the sample had long-standing problems and several prior treatment attempts, and may not be representative of a wider trauma-exposed clinical population. Also, the lack of assessment of functional impairment restricts the interpretation of our results, as the extent to which the endorsed problems affect daily life function is unknown. Although a full ITQ score was available from three out of four participants in our sample, six PTSD items were collected from corresponding items in the PCL-5 to create a full ITQ score for the remaining participants. These PCL-5 items are reported on the same scale and are highly similar in wording, but not identical, to the original PTSD items in the ITQ, which may have influenced our findings.

## Notes

1. Re-experiencing symptoms were represented by PCL-5 items 2 and 3 (corresponding to items 1 and 2 of the ITQ), avoidance by items 6 and 7 (corresponding to items 3 and 4 of the ITQ), and sense of threat by items 17 and 18 (corresponding to items 5 and 6 of the ITQ).

2. A parallel G-study treating re-experiencing, avoidance and sense of current threat as separate fixed facets, and the DSO symptoms as one fixed facet, is presented in Table A3 in the Appendix.

## Appendix

**Table A1.** Frequency of symptom endorsement (items ≥ 2), mean score and standard deviation for each of the 22 symptoms in the preliminary International Trauma Questionnaire (ITQ).

	Symptom endorsed Valid % (*n*)	Mean (SD)
PTSD symptoms		
Upsetting dreams (Re1)*	74.3 (150)	2.30 (1.27)
Reliving the event in the here and now (Re2)*	69.8 (141)	2.23 (1.22)
Internal avoidance (Av1)*	88.6 (179)	2.90 (1.05)
External avoidance (Av2)*	86.6 (175)	2.85 (1.05)
Being on guard (Th1)*	84.7 (171)	2.82 (1.19)
Jumpy/startled (Th2)*	79.2 (160)	2.71 (1.20)
DSO symptoms		
Intense reactions (AD1)	78.2 (158)	2.47 (1.06)
Long time to calm down (AD2)*	78.2 (158)	2.52 (1.04)
Feelings easily hurt (AD3)	82.7 (167)	2.72 (1.15)
Uncontrollable anger (AD4)	33.2 (67)	1.16 (1.20)
Reckless behaviour (AD5)	18.8 (38)	0.67 (0.99)
Numb (AD6)*	71.3 (144)	2.35 (1.22)
Difficulty feeling pleasure (AD7)	68.8 (139)	2.24 (1.23)
World is distant (AD8)	63.9 (129)	2.12 (1.31)
Feeling outside of body (AD9)	55.9 (113)	1.84 (1.43)
Failure (NSC1)*	81.2 (164)	2.87 (1.22)
Worthless (NSC2)*	78.7 (159)	2.76 (1.33)
Self-shame (NSC3)	84.2 (170)	2.97 (1.21)
Guilt (NSC4)	84.7 (171)	2.94 (1.14)
Cut off from others (DR1)*	77.2 (156)	2.37 (1.18)
Difficult to stay close to others (DR2)*	78.2 (158)	2.60 (1.22)
Avoiding relationships (DR3)	67.8 (137)	2.19 (1.23)

PTSD, post-traumatic stress disorder; DSO, disturbances in self-organisation; Re, re-experiencing; Av, avoidance; Th, sense of current threat; AD, affective dysregulation; NSC, negative self-concept; DR, disturbances in relationships. *Items included in the 12-item ITQ version.

**Table A2.** Standardized factor loadings (SE) for model 2.

Item	Re	Av	Th	Hyper	Deact	NSC	DR
Re1 – upsetting dreams	.720 (.062)						
Re2 – reliving the event	.766 (.068)						
Av1 – internal avoidance		.708 (.057)					
Av2 – external avoidance		.920 (.070)					
Th1 – being on guard			.878 (.038)				
Th2 – jumpy/startled			.791 (.041)				
AD1 – intense reactions				.738 (.052)			
AD2 – long time to calm down				.631 (.054)			
AD3 – feelings easily hurt				.581 (.062)			
AD4 – uncontrollable anger				.511 (.068)			
AD5 – reckless behaviour				.286 (.086)			
AD6 – numb					.748 (.036)		
AD7 – difficulty feeling pleasure					.787 (.039)		
AD8 – world is distant					.760 (.036)		
AD9 – feeling outside of body					.741 (.040)		
NSC1 – failure						.948 (.018)	
NSC2 – worthless						.948 (.013)	
NSC3 – shame						.934 (.015)	
NSC4 – guilt						.864 (.026)	
DR1 – cut off from others							.890 (.027)
DR2 – difficult to stay close to others							.812 (.033)
DR3 – avoiding relationships							.803 (.030)
**Second-order factor loadings**			**PTSD**			**DSO**	
Re-experiencing (Re)			.680 (.064)				
Avoidance (Av)			.610 (.072)				
Sense of current threat (Th)			.881 (.056)				
Affect hyperactivation (AD-H)						.817 (.047)	
Affect deactivation (AD-D)						.872 (.031)	
Negative self-concept (NSC)						.724 (.044)	
Disturbances in relationships (DR)						.884 (.031)	

Re, re-experiencing; Av, avoidance; Th, sense of current threat; AD, affective dysregulation; NSC, negative self-concept; DR, disturbances in relationships; Hyper, hyperactivation; Deact, deactivation; PTSD, post-traumatic stress disorder; DSO, disturbances in self-organisation.

All factor loadings are significant (*p* < .01).

**Table A3.** Estimated G-study variance and covariance components for 22-item International Trauma Questionnaire based on *p* × *i* design (*N* = 165).

Source	Re-experiencing	Avoidance	Sense of current threat	Disturbances in self-organization
Persons (*p*)	.76685	.64072	.83622	.49061
Items (*i*)	−.00251	−.00288	.00710	.42160
Persons by items (*pi*)	.71766	.47864	.57472	.42160
Relative contribution to composite universe score	7.44%	6.00%	9.88%	76.68%
